# Ulinastatin promotes macrophage efferocytosis and ameliorates lung inflammation via the ERK5/Mer signaling pathway

**DOI:** 10.1002/2211-5463.13461

**Published:** 2022-07-11

**Authors:** Jinju Li, Rongge Shao, Qiuwen Xie, Ke Qin, ShaoPeng Ming, Yongguo Xie, XueKe Du

**Affiliations:** ^1^ Department of Anesthesiology The Second Affiliated Hospital of Guangxi Medical University Nanning China; ^2^ Guangxi Key Laboratory of Basic Research on Perioperative Organ Function Injury and Prevention Nanning China; ^3^ Guangxi Clinical Research Center for Anesthesiology Nanning China; ^4^ Guangxi Engineering Research Center for Tissue & Organ Injury and Repair Medicine Nanning China

**Keywords:** efferocytosis, ERK5, lung inflammation, macrophage, Mer, ulinastatin

## Abstract

Acute lung injury (ALI) is a pneumonic response characterized by neutrophil infiltration. Macrophage efferocytosis is the process whereby macrophages remove apoptotic cells, and is required for ALI inflammation to subside. The glycoprotein ulinastatin (UTI) has an anti‐inflammatory effect during the acute stages of ALI, but its effect on efferocytosis and the subinflammatory stage of ALI is unclear. Extracellular signal‐regulated kinase 5 (ERK5) is a key protein in efferocytosis, and we thus hypothesized that it may be activated by UTI to regulate efferocytosis and the resolution of pneumonia. To test this hypothesis, here we monitored phagocytosis of macrophages through *in vivo* and *in vitro* experiments. Pulmonary edema, neutrophil infiltration, protein exudation, and inflammatory factor regression were observed on days 1, 3, 5, and 7 *in vivo.* RAW264.7 cells were pretreated with different concentrations of UTI and ERK5 inhibitors, and the expression of tyrosine‐protein kinase Mer (Mer) protein on macrophage membrane was detected. UTI increased the phagocytosis of apoptotic neutrophils by macrophages *in vitro* and *in vivo*, and promoted the resolution of pneumonia. The protein expression of ERK5 and Mer increased with UTI concentration, while the expression of Mer was down‐regulated by ERK5 inhibitors. Therefore, our results suggest that UTI enhances efferocytosis and reduces lung inflammation and injury through the ERK5/Mer signaling pathway, which may be one of the targets of UTI in the treatment of lung injury.

## Graphical abstract

Ulinastatin (UTI) has an anti‐inflammatory effect during the acute stage of ALI, but its effect on efferocytosis and the subinflammatory stage of ALI is unclear. ERK5 is a key protein in efferocytosis, it is unknown whether it is activated by UTI to regulate resolution of efferocytosis and pneumonia. Our study suggests that UTI enhances efferocytosis and reduces lung inflammation and injury through the ERK5/Mer signaling pathway.

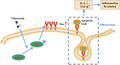

**Abbreviations**
ALI: acute lung injury
ERK5: extracellular signalregulated kinase 5
Mer: tyrosine‐protein kinase Mer
UTI: ulinastatin

Acute lung injury (ALI) is a critical clinical illness of common occurrence and is a pneumonic reaction characterized by neutrophil infiltration [1]. The pathophysiological process of ALI can be roughly divided into the acute inflammatory stage and the subsiding inflammatory stage. In the subsiding phase of inflammation, macrophages directly engulf and clear apoptotic neutrophils to repair damaged tissues, a process called efferocytosis, which promotes the recovery of lung epithelial and endothelial functions and the reconstruction of lung tissue structure. If apoptotic cells are not cleared in time, many intracellular danger signaling molecules are released by secondary cell necrosis, which further hinders tissue repair. Therefore, promoting the regression of pneumonia will shorten the pathophysiological process of ALI and reduce lung injury [2, 3].

Ulinastatin (UTI), a commonly used drug in clinical practice, is an endogenous molecule with actions against infectious pathogens and its pharmacological effects include inhibiting the excessive release of inflammatory mediators scavenging oxygen‐free radicals and alleviating lung injury by means of its anti‐inflammatory effect [4]. Xu et al. [5] showed that Xubijing combined with UTI was an effective sepsis treatment. Zhu et al. [6] found that UTI reduced doxorubicin‐induced myocardial injury in SD rats. However, the effect of UTI on the inflammatory resolution period following ALI remains unclear. In view of the critical role of macrophages, especially efferocytosis, in inflammation regression [7, 8], we investigated the effect of UTI on efferocytosis.

ERK5 is a member of the MAPK family [9, 10]. It can be activated by a series of stimuli (including pro‐inflammatory stimuli), transmitting extracellular stimulation signals to the intracellular, compartment, and having anti‐inflammatory and immunomodulatory functions [11, 12]. In addition, it has been reported that activation of ERK5 enhances macrophage efferocytosis, thus inhibiting the occurrence of atherosclerosis [13]. These pieces of evidence suggest that ERK5 is a key factor in the regulation of macrophage function. CD36 and Mer receptor tyrosine kinases on the cell membrane of macrophages recognize apoptotic cells via bridging proteins [14, 15, 16]. We previously demonstrated that isoflurane promotes the phagocytosis of apoptotic neutrophils by macrophages via AMPK‐mediated ADAM17/Mer signaling pathway [17]. Therefore, we investigated whether UTI regulates Mer receptors through activation of ERK5 and hence efferocytosis through Mer‐receptor tyrosine kinases.

This study aimed to determine the effect of UTI on macrophage efferocytosis in the context of ALI. There was a particular, focus on whether UTI affected efferocytosis through the ERK5/Mer signaling pathway, promotes pneumonia resolution, and alleviated lung inflammation and injury.

## Materials and methods

### Animals and laboratory protocols

Male c57bl/6 mice (8 weeks old and weighing 20 ± 2 g; Nanning, Guangxi, China) were purchased from the Experimental Animal Center of Guangxi Medical University (animal certificate number: SYXK9 (GUI) 2014‐0003). All animal surgeries were approved by the Ethics Committee of the Experimental Animal Center of Guangxi Medical University (Nanning, China; Approval No. 202008010). In the experimental design, we achieved the minimum necessary amount by optimizing the experimental scheme. In the process of the experiment, we used a relatively mild injection technique and the appropriate amount of adjuvant to reduce the serious side effects on animals and reduce their pain of the animals. Animals were assigned to one of four groups and treated as follows: LPS: 5 mg·kg^−1^ intravenous LPS; LPS + UTI: 5 mg·kg^−1^ intravenous LPS immediately followed by 50 000 U·kg^−1^ intraperitoneal UTI; Control group; UTI: 50 000 U·kg^−1^ intraperitoneal UTI. We used ERK5 inhibitor BIX02189 to re‐divide the animal experiments into the control group, BIX02189 group, LPS + UTI group, and LPS + UTI + BIX02189 group. BIX02189 group and LPS + UTI + BIX02189 group were intraperitoneally injected with 10 mg·kg^−1^ BIX02189 2 h before modeling. In addition, we used XMD8‐92 to divide mice into the CON group, XMD8‐92 group, LPS + UTI group, and LPS + UTI + XMD8‐92 group. Mice in the XMD8‐92 group and LPS + UTI + XMD8‐92 group were pre‐injected with XMD8‐92(10 mg·kg^−1^) once a week in advance [18]. Lung tissue and bronchial alveolar lavage fluid (BALF) were collected on days 1, 3, 5, and 7 after LPS‐modeling.

### Reagents and antibodies

UTI (Tempu, Guangzhou, China) was purchased from Guangdong Tempu Biochemical Pharmaceutical Co., LTD. LPS (Sigma, L2880, St. Louis, MO, USA) and PKH26 Red (Sigma, SLF‐MINI26) were purchased from Sigma Biochemical Corporation. CellTracker™ green B0DIPY™ (Invitrogen, C2102, California, USA) was purchased from Invitrogen. The ELISA kit (Huamei, Wuhan, China) was purchased from Wuhan Huamei Biological Engineering Co., LTD. BCA test kit (Soleibao, Beijing, China) and Diff‐Quick dye solution (Soleibao) were purchased from Beijing Soleibao Technology Co., LTD. BIX02189 was purchased from MedChemExpress (HY‐12056, New Jersey, USA). DMEM, RPMI1640, and FBS were purchased from Gibco BRL (Gaithersburg, MD, USA). Antibodies raised against the following proteins were used: ERK5 (1 : 1000, Abcam, Cambridge, USA, ab40809); Mertk (1 : 100, Abclonal, Wuhan, China, A5443); Goat anti‐rabbit immunoglobulin horseradish peroxidase (igg‐hrp; 1 : 30 000, Ray antibody, RM3003); Fluorophile conjugated goat anti‐rabbit secondary antibody (1 : 1000, Alexa Fluor 488; Invitrogen: MA700184A488).

### Cell culture

Mouse peritoneal macrophage cell‐line, RAW264.7 (Procell, Wuhan, China), and human acute myeloid leukemia cell‐line, HL60 (Procell), were purchased from Wuhan Procell Life Science and Technology Co., LTD. RAW264.7 cells were cultured in high glucose DMEM supplemented with 10% (v/v) FBS and HL60 cells were cultured in RPMI 1640 medium supplemented with 12% (v/v) FBS, 100 U·mL^−1^ penicillin, and 100 μg·mL^−1^ streptomycin and in humidified air with 5% CO_2_ at 37 °C. UTI was dissolved in PBS and further diluted with cell culture media.

### Specimen collection

The left lung was isolated immediately after anesthesia and fixed with 4% paraformaldehyde. The upper lobe of the right lung was isolated, weighed, and baked at 60 °C for 72 h. Bronchoalveolar lavage fluid (BALF) was collected into 2 mL pre‐cooled phosphate‐buffered saline (PBS) and the total number of cells was determined immediately. BALF was centrifuged and the supernatant was stored at −80 °C for determination of total protein and inflammatory factor levels. The cell pellet was immediately resuspended in PBS and numbers of neutrophils were determined by Diff‐Quick staining solution (Soleibao).

### Lung histopathological analysis

The left lung was dehydrated, embedded, sectioned, stained with hematoxylin–eosin (HE; Soleibao) and histopathological changes were observed under light microscopy (Olympus, Tokyo, Japan), as previously described [19, 20]. Lung tissue was assigned an injury score, as follows: 0: normal; 1: mild injury affecting 25% of lung tissue; 2: moderate injury affecting 25–50% of lung tissue; 3: severe injury affecting 50–75% of lung tissue; 4: very severe injury affecting more than 75% of lung tissue.

### Inflammatory response

A wet‐dry ratio was calculated for the lung tissue to indicate lung exudation during LPS‐induced injury. The wet weight was determined immediately after collection and tissue were dried in an oven for 72 h before determination of the dry weight. BALF cells were counted by hemocytometer and concentrations of protein plus inflammatory factors were measured with a BCA test kit (Soleibao). The BALF cell pellet was re‐suspended in PBS and cells were evenly distributed on a glass slide using a cell slicer before estimation of neutrophil number by Diff‐Quick dye staining, as previously described.

### Phagocytosis by alveolar macrophages *in vivo*

Bronchial alveolar lavage fluid was centrifuged and cell pellets resuspended in PBS with 30% FBS to a density of 5 × 10^5^ cells·mL^−1^, as described previously. Two hundred microliters cell suspension were added to each sling hole and evenly distributed on the slide with cypres (MOTIC, Xiamen, China). Diff‐Quick dye was added, according to the manufacturer's instructions [17]. After staining, 300 macrophages were randomly selected under the microscope, and number of apoptotic bodies within the macrophages were divided by 300 to obtain the phagocytic rate.

### Preparation of apoptotic cells

HL60 cells were suspended in 10 mL RPMI1640 medium and seeded into a Petri dish, 9 cm from the ultra‐clean cell for 30 min UV irradiation. After incubation at 37 °C for 3 h, apoptotic HL60 cells were stained with PKH26 Red (Sigma, SLF‐MINI26).

### Macrophage phagocytosis *in vitro*

*In vitro* phagocytosis was assessed as described previously [17]. Briefly, RAW264.7 cells were seeded into 24‐well plates at 10^5^ cells·mL^−1^. After adhesion, cells were pretreated with either 1000 or 5000 U·mL^−1^ UTI for 3 h [21]. Cells were stained with CellTracker™ Green B0DIPY™ (Invitrogen, C2102) dye to a final concentration of 5 μm for 30 min. Cells were washed three times with PBS and 10^6^ cells per well apoptotic HL60 cells were added with incubation in a cell incubator overnight. Wells were washed three times with PBS and an anti‐fluorescence quench agent added. Phagocytosis was observed under a fluorescence microscope (Olympus).

### Immunofluorescence

The cell experiment was first divided into 0, 1000, and 5000 U·mL^−1^ groups. Mouse peritoneal macrophages RAW264.7 cells were seeded into 24 well plates at 10^5^·mL^−1^. After cell adherence, the cells were pretreated with ulinastatin at different concentrations for 3 h. ERK5 inhibitor BIX02189 (MedChemExpress, HY‐12056) was used to divide the cells into blank control group, BIX02189 group, 5000 U·mL^−1^ group, and 5000 U·mL^−1^ + BIX02189 group. The cells were pretreated with ERK5 inhibitor BIX02189 (15 μm)for 1 h and then 5000 U·mL^−1^ UTI for 3 h. In addition, ERK5 inhibitor XMD8‐92 (Selleck, S7525, Shanghai, China) was used to divide the cells into control group, XMD8‐92 group, 5000 U·mL^−1^ group, and 5000 U·mL^−1^ + XMD8‐92 group. The cells were pretreated with ERK5 inhibitor XMD8‐92 (10 μm) [22] for 3 h and then 5000 U·mL^−1^ UTI for 3 h. Cells were washed three times with PBS and 4% paraformaldehyde was added to fix the cells at 4 °C overnight. After washing three times with PBS, primary antibodies raised against Mer (1 : 100, Abclonal, A5443) were added for overnight incubation at 4 °C. Fluorescently conjugated secondary antibodies (1 : 1000, Alexa Fluor 488; Invitrogen: MA700184A488) were used for staining, and cells were incubated at room temperature in the dark for 1 h. After three washes with PBS, DAPI (Sigma‐Aldrich) was used for nuclear staining, slides observed by fluorescence microscope (Olympus) equipped with Nikon DS‐U3 imaging system, and fluorescence intensity quantified with image‐j software (NIH, New York, NY, USA).

### Western blotting

The cells were grouped as before. Total protein was extracted and protein loading buffer (Soleibao) was added. After boiling and denaturation, SDS/PAGE electrophoresis was carried out, proteins were transferred to a PVDF (ISEQ00010, Merck Millipore, Massachusetts, USA) membrane and incubated with ERK5 primary antibody (1 : 1000, Abcam, ab40809) at 4 °C overnight. Goat anti‐rabbit fluorescent secondary antibody (1 : 30 000, Ray antibody, RM3003) was added, incubated at room temperature for 1 h, and bands developed by fluorescence scanner. image‐j software (NIH) was used to determine the gray value of each band and relative expression levels of ERK5 protein represented by the ratio of the ERK5 gray band to the GAPPH band.

### Statistical analysis

spss 23.0 software (GraphPad Software, La Jolla, CA, USA) was used for statistical analysis. The student's *t*‐test was used to compare means ± standard deviations (*x* ± s), for two groups with normal distribution and ANOVA and SNK test to compare multiple groups. A value of *P* < 0.05 was considered statistically significant.

## Results

### Ulinastatin enhanced the macrophage efferocytosis of RAW264.7 cells and alveolar macrophages on apoptotic neutrophils in mice

**Fig. 1:**
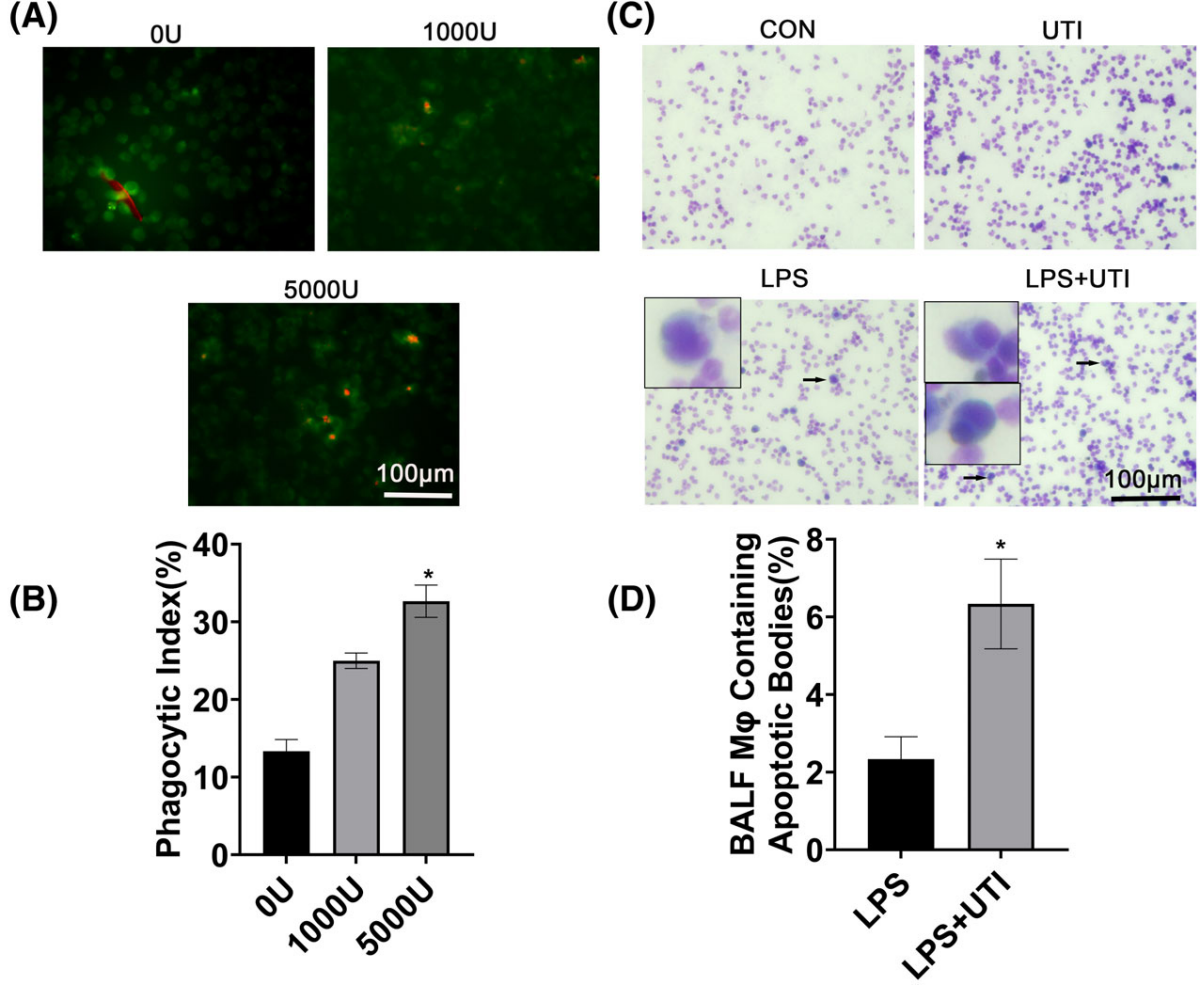
Ulinastatin (UTI) enhanced macrophage efferocytosis. (A) Effect of UTI on RAW264.7 cell phagocytosis and apoptosis of HL60 cell. Representative fluorescence image of HL60 phagocytotic apoptosis in RAW264.7 cells. Scale: 100 μm; (B) phagocytic index based on fluorescence image. (C) UTI enhanced the ability of alveolar macrophages to phagocytose apoptotic PMNs. Representative images of PMNs from alveolar macrophage phagocytic apoptosis, scale:100 μm; (D) phagocytic index based on representative images. Arrows indicate macrophages containing apoptotic bodies. Data are expressed as mean ± standard deviation, one‐way ANOVA, *n* = 3. *P < 0.05 vs. CON Group or 0 U·mL^−1^ UTI group. [Colour figure can be viewed at wileyonlinelibrary.com]

The process of macrophage phagocytosis and clearance of apoptotic neutrophils is the key to the resolution of pneumonia [23]. Here, the effect of UTI on macrophage efferocytosis was investigated. A dose‐dependent increase in phagocytosis of RAW264.7 cells in the range: 0, 1000, and 5000 U·mL^−1^ UTI was observed (Fig. 1A,B). In a mouse model of LPS‐induced lung injury, the phagocytosis of alveolar macrophages in alveolar lavage fluid in the LPS + UTI group was higher than that in the LPS and CON groups (Fig. 1C,D). These results suggest that UTI enhanced the cellular burial of macrophages. The phagocytosis of RAW264.7 cells increased in a dose‐dependent manner when pretreated with 0, 1000, and 5000 U·mL^−1^ UTI.

### Ulinastatin ameliorated LPS‐induced lung injury by enhancing macrophage efferocytosis

**Fig. 2:**
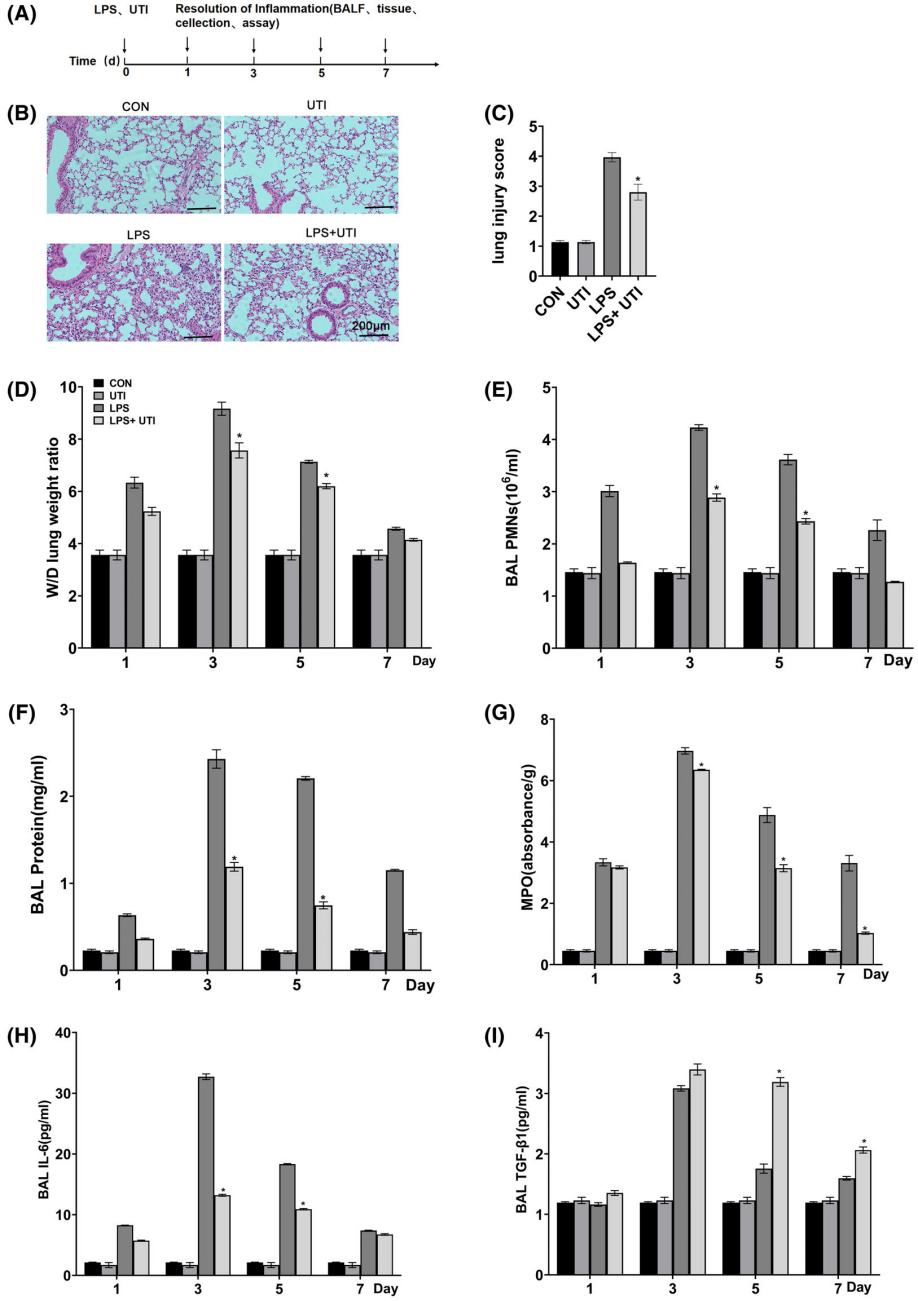
LPS induced morphological damage in c57bj/6 mice, and pulmonary edema and inflammation in CON, UTI, LPS, LPS + UTI groups. (A) Experimental protocols of induction and time course of resolution of lung inflammation post‐LPS or vehicle (CON) challenge in wild‐type mice. (B) Hematoxylin and eosin (H&E) staining of mouse lung tissue, scale: 200 μm. (C) The pathological score was assessed by H&E staining. (D) Levels of neutrophils in BALF. (E) Total protein concentration in BALF. (F) Lung tissue wet‐dry ratio. (G) Level of MPO in BALF. (H) IL‐6 levels in BALF. (I) The level of TGF‐β in BALF. Data were expressed as mean ± standard deviation, one‐way ANOVA, *n* = 3. *P < 0.05 vs. CON Group or 0 U·mL^−1^ UTI group.

Since UTI enhanced macrophage efferocytosis, the possibility of UTI modulating efferocytosis to accelerate the resolution of pneumonia in LPS‐induced lung injury models was investigated (Fig. 2A). After LPS infusion, lung pathology (Fig. 2B,C), pulmonary edema (Fig. 2D), PMN count in BALF fluid (Fig. 2E), protein level (Fig. 2F), the levels of MPO (Fig. 2G), IL‐6 (Fig. 2H), and TGF‐β1 (Fig. 2I) increased, peaked on day 3, and then gradually decreased. UTI was administered immediately after LPS infusion, resulting in reduced lung tissue damage, and pulmonary edema, neutrophil infiltration, protein exudation, and inflammatory factor regression were accelerated on days 5 and 7. Our results suggest that UTI ameliorated LPS‐induced lung injury by enhancing macrophage efferocytosis.

### Ulinastatin upregulated surface expression of Mer receptor tyrosine kinase in macrophages

**Fig. 3:**
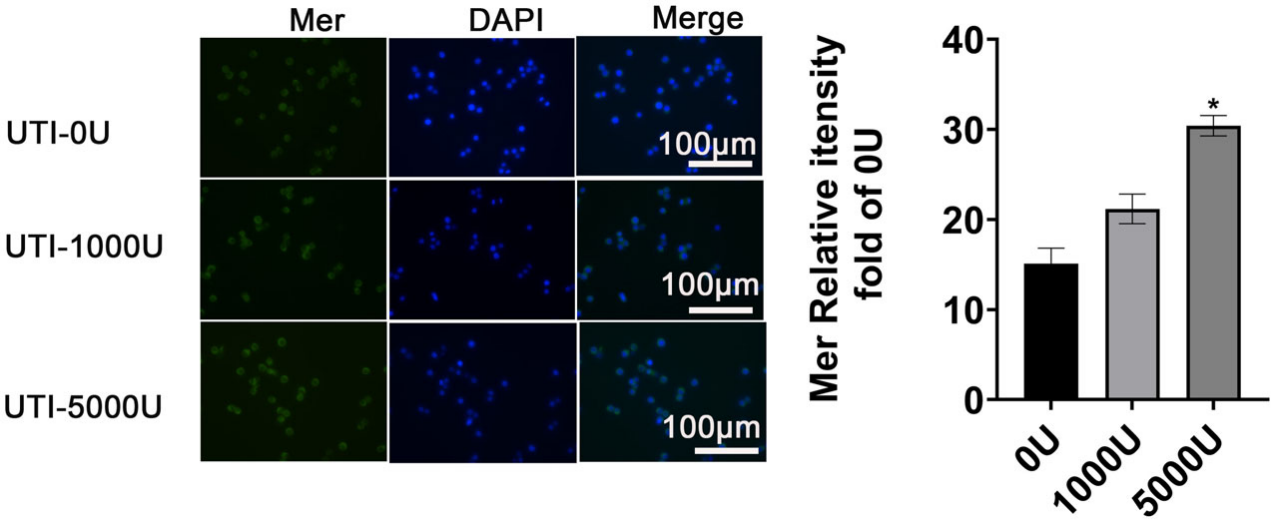
Ulinastatin increased surface expression of Mer protein. On the left, Mer protein level was detected by immunofluorescence assay. Representative fluorescence image of protein expression; on the right, the relative protein expression of Mer. Data are expressed as mean ± standard deviation, one‐way ANOVA, *n* = 3. *P < 0.05 vs. 0 U·mL^−1^ UTI group. [Colour figure can be viewed at wileyonlinelibrary.com]

Mer receptor tyrosine kinases on macrophage cell membranes recognize apoptotic cells by means of bridging proteins, and the resolution of inflammation depends on the apoptosis and clearance of recruited neutrophils [14]. The regulation of Mer expression by UTI was investigated. Compared with the 0 U·mL^−1^ group, the surface expression of Mer in 1000 and 5000 U·mL^−1^ UTI groups significantly increased (Fig. 3). These results suggest that UTI upregulated Mer expression on the macrophage membrane surface.

### Ulinastatin upregulated Mer expression by activating ERK5

**Fig. 4:**
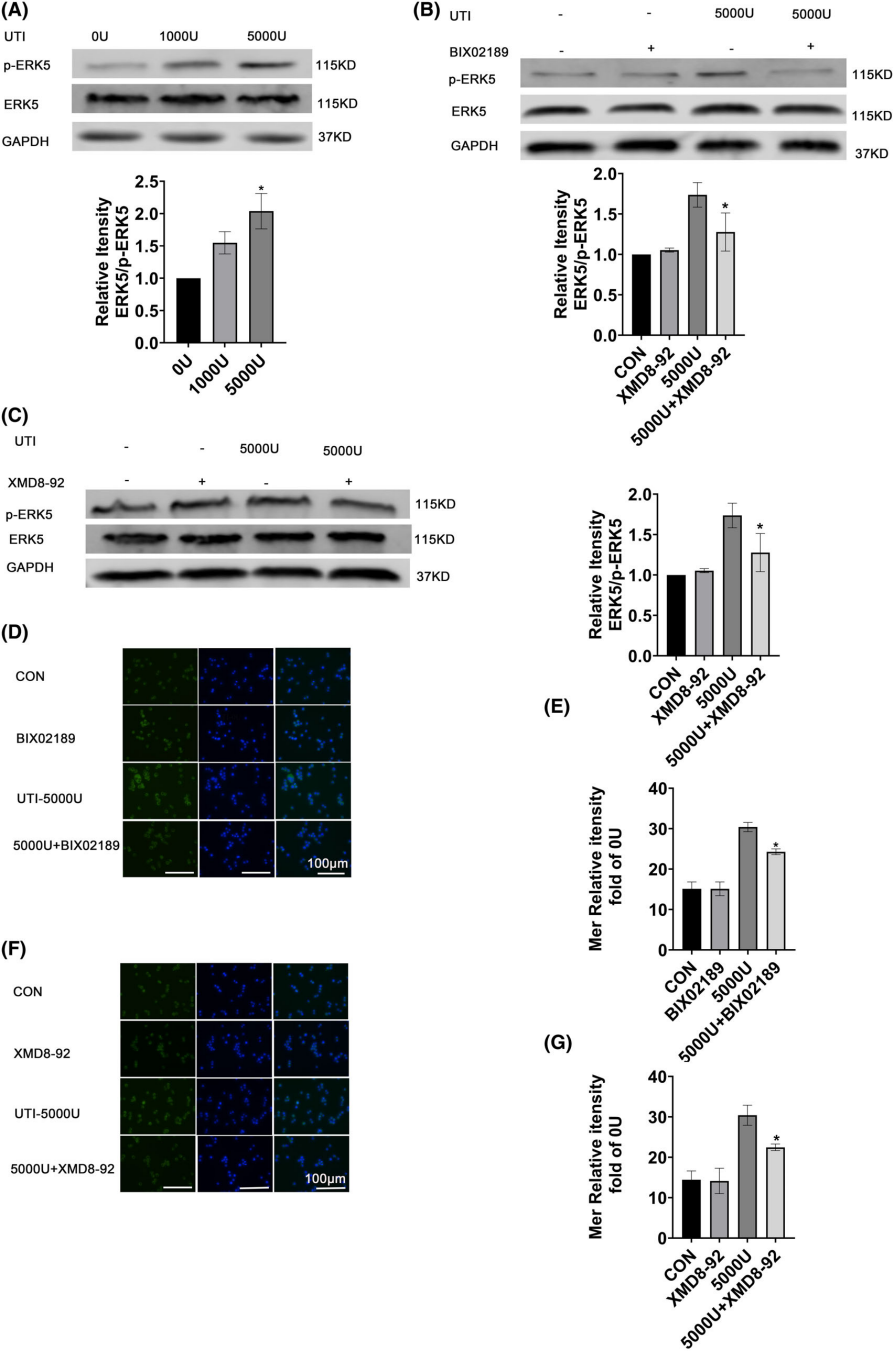
Ulinastatin upregulated Mer expression by activating ERK5. (A) ERK5 and p‐ERK5 protein levels were detected by Western blotting. Upper part: Representative blot of protein expression; lower part: Relative protein expression of ERK5 and p‐ERK5. (B) ERK5 and p‐ERK5 protein levels were determined by Western blotting after treatment with inhibitor BIX02189. Upper part: Representative blot of protein expression; lower part: Relative protein expression of ERK5 and p‐ERK5. (C) ERK5 protein levels were determined by Western blotting after treatment with the inhibitor XMD8‐92. Left: Representative blot of protein expression; right: Relative protein expression of ERK5 and p‐ERK5. (D) Immunofluorescence assay for the determination of membrane protein Mer after treatment with the inhibitor BIX02189. (E) Relative protein expression of membrane protein Mer. (F) The level of membrane protein Mer was determined by immunofluorescence assay after treatment with the inhibitor XMD8‐92. (G) Relative protein expression of membrane protein Mer. Data is expressed as mean ± standard deviation, one‐way ANOVA, *n* = 3, *P < 0.05 vs. 0 U·mL^−1^ UTI group. [Colour figure can be viewed at wileyonlinelibrary.com]

ERK5 is a serine protein kinase that is activated by phosphorylation and translocated from the cytoplasm to the nucleus, where it exerts anti‐inflammatory and immunomodulatory effects [9]. Therefore, p‐ERK5 activity was investigated to determine whether UTI enhanced macrophage efferocytosis by mediating Mer expression on the cell surface through modulation of p‐ERK5 activity. Compared with the 0 U·mL^−1^ group, the 1000 and 5000 U·mL^−1^ UTI groups had significantly increased expression of p‐ERK5 (Fig. 4A). The ERK5 inhibitor, BIX02189, inhibited the expression of p‐ERK5 (Fig. 4B), and the expression of Mer on the macrophage surface was also decreased (Fig. 4D,E). In addition, the use of another ERK5 inhibitor, XMD8‐92, show similar results (Fig. 4C) and decreased Mer expression with p‐ERK5 inhibition was consistently shown (Fig. 4F,G). In addition, we also examined the effect of ERK5 on Mer at the RNA level. The experimental results showed that there was no change in Mer RNA levels in groups 0, 1000, and 5000 U (Fig. S1A). Similarly, RNA levels of Mer in each group remained unchanged after treatment with ERK5 inhibitor (Fig. S1B,C). Therefore, we speculated that ERK5 might have no effect on the RNA level of Mer after UTI activation. These results suggest that ulinastatin upregulated Mer expression by activating ERK5.

### Ulinastatin enhanced macrophage efferocytosis and promoted pneumonia resolution through the ERK5/Mer pathway

**Fig. 5:**
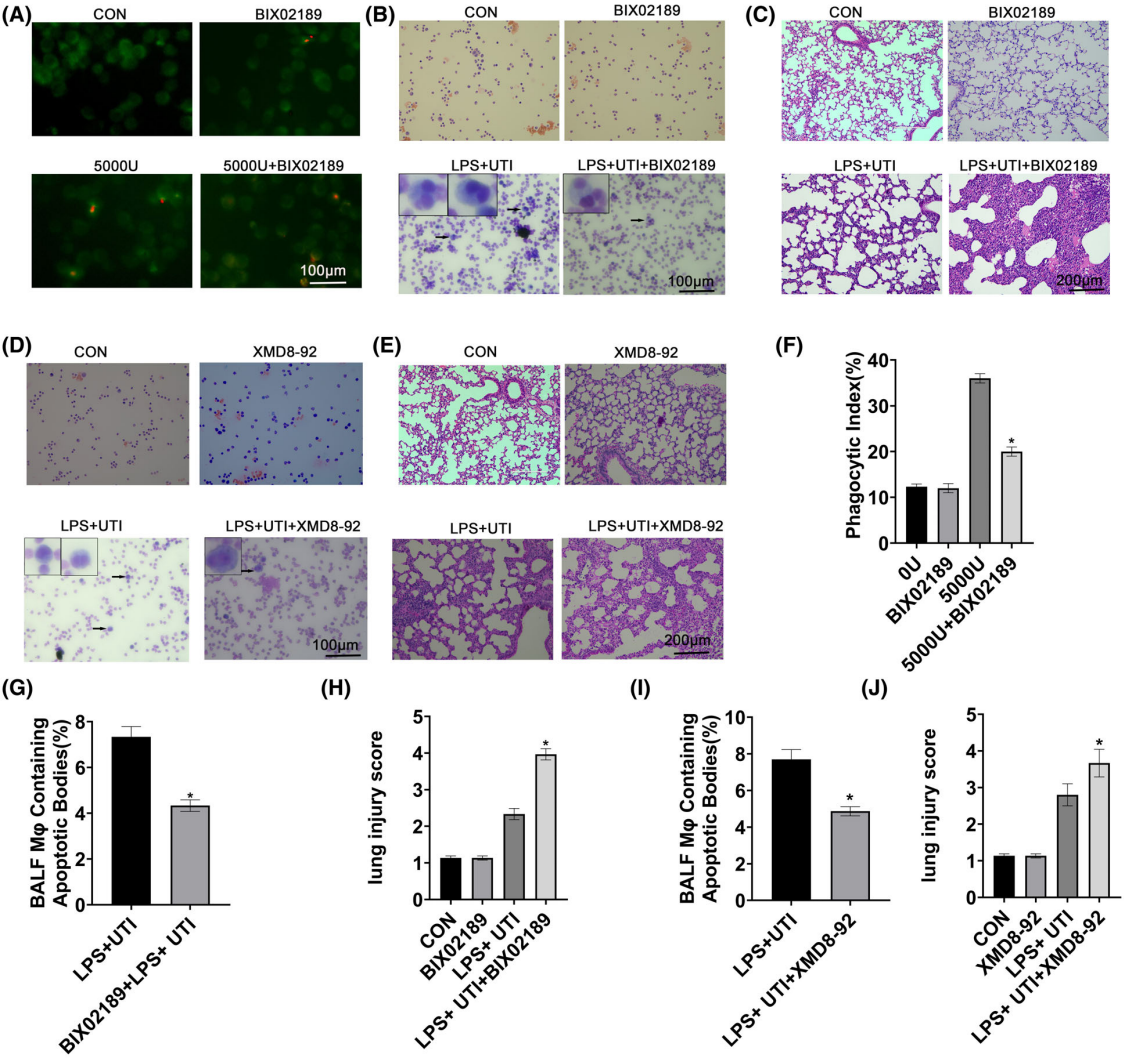
Ulinastatin enhanced macrophage efferocytosis and ameliorated LPS‐induced inflammatory injury. (A) Effect of UTI on phagocytosis and apoptosis of HL60 cells by RAW264.7 cells after using BIX02189. Left panel shows representative fluorescence images of HL60 phagocytic apoptosis in RAW264.7 cells. Scale, 100 μm; (B) UTI enhanced the ability of alveolar macrophage to phagocytose apoptotic PMNs after use of BIX02189. Representative images of apoptotic PMNs from alveolar macrophage phagocytizing cells, scale:100 μm; arrows indicate macrophages containing apoptotic bodies. (C) Hematoxylin and eosin (H&E) staining of mouse lung tissue after use of BIX02189, scale: 200 μm; (D) UTI enhanced the ability of alveolar macrophage to phagocytose apoptotic PMNs with XMD8‐92. Representative images of apoptotic PMNs from alveolar macrophages phagocytizing cells, scale:100 μm; arrows indicate macrophages containing apoptotic bodies. (E) Hematoxylin and eosin (H&E) staining of mouse lung tissue after use of XMD8‐92, scale: 200 μm; (F) Phagocytosis index (BIX02189) based on fluorescence images. (G) Phagocytosis index (BIX02189) based on representative images. (H) H&E staining was used to assess the pathological score (BIX02189). (I) Phagocytosis index (XMD8‐92) based on representative images. (J) H&E staining was used to assess the pathological score (XMD8‐92). Data are presented as mean ± standard deviation, one‐way ANOVA, *n* = 3. *P < 0.05 vs. UTI + LPS group or 5000 U·mL^−1^ UTI group. [Colour figure can be viewed at wileyonlinelibrary.com]

To further elucidate the critical role of ERK5 in macrophage efferocytosis, ERK5 inhibitors were used in *in vitro* and *in vivo* models. Compared with the 5000 U·mL^−1^ UTI group, the phagocytosis of RAW264.7 cells in the 5000 U·mL^−1^ + BIX02189 group was significantly reduced (Fig. 5A,F). In the LPS‐induced lung injury *in vivo* mouse model, the phagocytic capacity of alveolar macrophages in the bronchoalveolar lavage fluid of the LPS + UTI + BIX02189 group was reduced relative to that in the LPS + UTI group (Fig. 5B,G), and lung histopathological score was reduced (Fig. 5C,H). In addition, use of another ERK5 inhibitor, XMD8‐92, resulted in reductions of the phagocytic ability of alveolar macrophages in the LPS + UTI + BIX02189 group (Fig. 5D,I) and lung histopathology scores (Fig. 5E,J). In combination, these data suggest that UTI enhanced macrophage efferocytosis and promoted the resolution of pneumonia through the ERK5/Mer pathway.

## Discussion

This study investigated the effect of UTI on macrophage efferocytosis and the role of the ERK5/Mer pathway during deactivation of ALI inflammation *in vivo* and *in vitro*. Our study confirmed that UTI significantly enhanced macrophage efferocytosis of apoptotic cells and accelerated the resolution of LPS‐induced pneumonia. UTI upregulated Mer expression on the macrophage surface by activating ERK5. Inhibition of ERK5 reduced macrophage phagocytosis, slowed the process of pneumonia resolution, and reduced Mer expression on the macrophage surface. Our results suggest that UTI enhances macrophage endocytosis through the ERK5/Mer signaling pathway, thereby accelerating the resolution of pneumonia.

UTI is a serine protease inhibitor with anti‐inflammatory and immunomodulatory roles in a variety of inflammatory diseases [24, 25]. Ju et al. [26] found that UTI improved lung inflammation and injury in rats by inhibiting neutrophil activation, reducing inflammatory cell infiltration, and downregulating inflammatory cytokines. Similarly, Lu et al. [27] showed that UTI inhibited LPS‐induced activation of the TLR4/MyD88/NF‐κB signaling pathway, thereby reducing inflammation and preventing lung injury. Moreover, Hang et al. [28] found that UTI exerted lung protection by up‐regulating the expression of aquaporin in a porcine acute respiratory distress syndrome two‐hit model. In addition, UTI stabilizes the lysosomal membrane, inhibits the production of myocardial inhibitory factors, inhibits the release of lysosomal enzymes, scavenges oxygen‐free radicals, inhibits the excessive release of inflammatory mediators, improves human microcirculation and tissue perfusion, and plays a role in the function of tissues and organs. There are all effects, Which contribute to the protection of tissues against damage. These studies suggest that UTI may play an important role in lung injury. During the inflammation regression phase of ALI, the inflammation process depends on the apoptosis and clearance of recruited neutrophils. Regulatory mechanisms of macrophage efferocytosis directly affect the recognition, engulfment, and digestion of apoptotic cells, thereby affecting the progression of pneumonic resolution. Therefore, whether UTI promoted recovery from lung injury by accelerating the resolution of ALI inflammation was investigated during this study. These results provide evidence that UTI accelerates the resolution of pneumonia by enhancing macrophage efferocytosis.

Our previous study confirmed that a signaling pathway involving Mer receptors enhances isoflurane‐induced macrophage efferocytosis [17], and this study, found that with the increasing UTI concentration, the expression of Mer receptors on the macrophages surface increased. However, it is not clear how UTI mediates Mer receptor tyrosine kinases to enhance efferocytosis [29]. Luiz et al. [29] found that the MEK5/ERK5 signaling pathway mediates IL‐4‐induced differentiation of M2 macrophages by regulating c‐MYC expression. Giurisato et al. [30] found that extracellular regulation of p21 expression mediated by protein kinase 5 promotes macrophage proliferation and tumor growth and metastasis. These studies suggest that ERK5 may be a key factor in the regulation of macrophage function. Does UTI regulate Mer receptors by activating ERK5 and thus regulate macrophage efferocytosis? This study, found that ERK5 expression increased with increasing UTI concentration, and macrophage phagocytosis of apoptotic cells also increased. In the presense of ERK5 inhibitors, Mer expression decreased, and macrophage phagocytosis was also reduced. Furthermore, in the *in vivo* model of LPS‐induced lung injury, it was also found that alveolar macrophage phagocytic apoptosis phagocytic volume decreased in alveolar lavage fluid. Thus, and the pathological injury of lung tissue was aggravated. These results suggest that UTI enhances macrophage efferocytosis through the ERK5/Mer signaling pathway and promotes the resolution of pneumonia.

## Conclusion

In conclusion, UTI enhances macrophage efferocytosis through the ERK5/Mer signaling pathway, promotes the regression of pneumonia, alleviates lung injury, and provides a new target and theoretical basis for further development of more effective drugs to treat lung injury.

## Conflict of interest

The authors declare no conflict of interest.

## Author contributions

JL: conceptualization, methodology, writing—first draft; RS: writing review and editing; QX: investigation department, designed and interpreted the results; KQ: methods; collected materials and resources; SPM: data management, interpreted, and supervised the study; YX: resources department, conducted literature analysis; XKD: supervision, project management, fundraising. All authors read and approved the manuscript and scrutinized all data. The authors declare that all data were generated in‐house.

## Supporting information


**Fig. S1.** The effect of ERK5 on Mer at the RNA level. (A) RNA levels of Mer in 0, 1000 and 5000 U·mL^−1^ groups. (B) RNA levels of Mer in 0 U·mL^−1^, BIX02189, 5000 U·mL^−1^ and 5000 U+ BIX02189 groups. (C) RNA levels of Mer in 0 U·mL^−1^, XMD8‐92, 5000 U·mL^−1^ and 5000 U+ XMD8‐92 groups.

## Data Availability

The data that support the findings of this study are available from the corresponding author (duxueke@gxmu.edu.cn) upon reasonable request.

## References

[1] Huang X , Xiu H , Zhang S , Zhang G . The role of macrophages in the pathogenesis of ALI/ARDS. Mediators Inflamm. 2018;2018:1264913. 29950923 10.1155/2018/1264913 PMC5989173

[2] Butt Y , Kurdowska A , Allen TC . Acute lung injury: a clinical and molecular review. Arch Pathol Lab Med. 2016;140:345–50. 27028393 10.5858/arpa.2015-0519-RA

[3] Mowery NT , Terzian WTH , Nelson AC . Acute lung injury. Curr Probl Surg. 2020;57:100777. 32505224 10.1016/j.cpsurg.2020.100777

[4] Atal SS , Atal S . Ulinastatin – a newer potential therapeutic option for multiple organ dysfunction syndrome. J Basic Clin Physiol Pharmacol. 2016;27:91–9. 26565549 10.1515/jbcpp-2015-0003

[5] Xu Y , Zhong H , Liu W , Du J , Han W . Xuebijing combined with Ulinastatin for treating septic patients. Am J Emerg Med. 2021;45:525–6. 32703606 10.1016/j.ajem.2020.07.015

[6] Zhu JG , Jin K , Ren Y . Ulinastatin reduces myocardial injury induced by doxorubicin in SD rats. Eur Rev Med Pharmacol Sci. 2020;24:10769–78. 33155237 10.26355/eurrev_202010_23437

[7] Wang LX , Zhang SX , Wu HJ , Rong XL , Guo J . M2b macrophage polarization and its roles in diseases. J Leukoc Biol. 2019;106:345–58. 30576000 10.1002/JLB.3RU1018-378RR PMC7379745

[8] Yunna C , Mengru H , Lei W , Weidong C . Macrophage M1/M2 polarization. Eur J Pharmacol. 2020;877:173090. 32234529 10.1016/j.ejphar.2020.173090

[9] Sun Y , Liu WZ , Liu T , Feng X , Yang N , Zhou HF . Signaling pathway of MAPK/ERK in cell proliferation, differentiation, migration, senescence and apoptosis. J Recept Signal Transduct Res. 2015;35:600–4. 26096166 10.3109/10799893.2015.1030412

[10] Cargnello M , Roux PP . Activation and function of the MAPKs and their substrates, the MAPK‐activated protein kinases. Microbiol Mol Biol Rev. 2011;75:50–83. 21372320 10.1128/MMBR.00031-10 PMC3063353

[11] Wang Y , Zhang X , Gao L , Li J , Chen W , Chi J , et al. Cortistatin exerts antiproliferation and antimigration effects in vascular smooth muscle cells stimulated by ang II through suppressing ERK1/2, p38 MAPK, JNK and ERK5 signaling pathways. Ann Transl Med. 2019;7:561. 31807542 10.21037/atm.2019.09.45 PMC6861776

[12] Gámez‐García A , Bolinaga‐Ayala I , Yoldi G , Espinosa‐Gil S , Diéguez‐Martínez N , Megías‐Roda E , et al. ERK5 inhibition induces autophagy‐mediated cancer cell death by activating ER stress. Front Cell Dev Biol. 2021;9:742049. 34805151 10.3389/fcell.2021.742049 PMC8600073

[13] Heo KS , Cushman HJ , Akaike M , Woo CH , Wang X , Qiu X , et al. ERK5 activation in macrophages promotes efferocytosis and inhibits atherosclerosis. Circulation. 2014;130:180–91. 25001623 10.1161/CIRCULATIONAHA.113.005991 PMC4439099

[14] Wu H , Zheng J , Xu S , Fang Y , Wu Y , Zeng J , et al. Mer regulates microglial/macrophage M1/M2 polarization and alleviates neuroinflammation following traumatic brain injury. J Neuroinflammation. 2021;18:2. 33402181 10.1186/s12974-020-02041-7 PMC7787000

[15] Li W , Xie L , Ma J , Cheng M , Fan L , Xu Y , et al. Gas 6 or Mer deficiency ameliorates silica‐induced autophagosomes accumulation in mice lung. Toxicol Lett. 2021;337:28–37. 33232774 10.1016/j.toxlet.2020.11.013

[16] Zhen Y , Finkelman FD , Shao WH . Mechanism of Mer receptor tyrosine kinase inhibition of glomerular endothelial cell inflammation. J Leukoc Biol. 2018;103:709–17. 29350876 10.1002/JLB.3A0917-368R PMC6150462

[17] Du X , Jiang C , Lv Y , Dull RO , Zhao YY , Schwartz DE , et al. Isoflurane promotes phagocytosis of apoptotic neutrophils through AMPK‐mediated ADAM17/Mer signaling. PLoS ONE. 2017;12:e0180213. 28671983 10.1371/journal.pone.0180213 PMC5495389

[18] Howell SJ , Lee CA , Batoki JC , Zapadka TE , Lindstrom SI , Taylor BE , et al. Retinal inflammation, oxidative stress, and vascular impairment is ablated in diabetic mice receiving XMD8‐92 treatment. Front Pharmacol. 2021;12:732630. 34456740 10.3389/fphar.2021.732630 PMC8385489

[19] Matute‐Bello G , Downey G , Moore BB , Groshong SD , Matthay MA , Slutsky AS , et al. An official American Thoracic Society workshop report: features and measurements of experimental acute lung injury in animals. Am J Respir Cell Mol Biol. 2011;44:725–38. 21531958 10.1165/rcmb.2009-0210ST PMC7328339

[20] D'alessio FR , Tsushima K , Aggarwal NR , Mock JR , Eto Y , Garibaldi BT , et al. Resolution of experimental lung injury by monocyte‐derived inducible nitric oxide synthase. J Immunol. 2012;189:2234–45. 22844117 10.4049/jimmunol.1102606 PMC3424351

[21] Li ST , Dai Q , Zhang SX , Liu YJ , Yu QQ , Tan F , et al. Ulinastatin attenuates LPS‐induced inflammation in mouse macrophage RAW264.7 cells by inhibiting the JNK/NF‐κB signaling pathway and activating the PI3K/Akt/Nrf2 pathway. Acta Pharmacol Sin. 2018;39:1294–304. 29323338 10.1038/aps.2017.143 PMC6289329

[22] Jin H , Shi Y , Lv Y , Yuan S , Ramirez CFA , Lieftink C , et al. EGFR activation limits the response of liver cancer to lenvatinib. Nature. 2021;595:730–4. 34290403 10.1038/s41586-021-03741-7

[23] Proto JD , Doran AC , Gusarova G , Yurdagul A Jr , Sozen E , Subramanian M , et al. Regulatory T cells promote macrophage efferocytosis during inflammation resolution. Immunity. 2018;49:666–77.e6. 30291029 10.1016/j.immuni.2018.07.015 PMC6192849

[24] Cao C , Yin C , Shou S , Wang J , Yu L , Li X , et al. Ulinastatin protects against LPS‐induced acute lung injury by attenuating TLR4/NF‐κB pathway activation and reducing inflammatory mediators. Shock. 2018;50:595–605. 29324628 10.1097/SHK.0000000000001104

[25] Fang S , Li P , Zhu C , Han X , Bao P , Guo W . Research progress of ulinastatin in the treatment of liver diseases. Int J Clin Exp Pathol. 2020;13:2720–6. 33284867 PMC7716140

[26] Ju M , He H , Chen S , Liu Y , Liu Y , Pan S , et al. Ulinastatin ameliorates LPS‐induced pulmonary inflammation and injury by blocking the MAPK/NF‐κB signaling pathways in rats. Mol Med Rep. 2019;20:3347–54. 31432172 10.3892/mmr.2019.10561

[27] Lu Q , Liu Z , He W , Chu X . Protective effects of ulinastatin on rats with acute lung injury induced by lipopolysaccharide. Bioengineered. 2021. 10.1080/21655979.2021.1987083 PMC10813561 34637694

[28] Hang CC , Guo YH , Li CS , Wang S . Ulinastatin exerts the protective effects of lung by up‐regulating aquaporins expression in a two‐hit porcine model of acute respiratory distress syndrome. Biomed Environ Sci. 2021;34:1029–32. 34981729 10.3967/bes2021.140

[29] Luiz JPM , Toller‐Kawahisa JE , Viacava PR , Nascimento DC , Pereira PT , Saraiva AL , et al. MEK5/ERK5 signaling mediates IL‐4‐induced M2 macrophage differentiation through regulation of c‐Myc expression. J Leukoc Biol. 2020;108:1215–23. 32745297 10.1002/JLB.1MA0520-016R

[30] Giurisato E , Lonardi S , Telfer B , Lussoso S , Risa‐Ebrí B , Zhang J , et al. Extracellular‐regulated protein kinase 5‐mediated control of p21 expression promotes macrophage proliferation associated with tumor growth and metastasis. Cancer Res. 2020;80:3319–30. 32561530 10.1158/0008-5472.CAN-19-2416 PMC7611207

