# Supplementary material for: Ulinastatin promotes macrophage efferocytosis and ameliorates lung inflammation via the ERK5/Mer signaling pathway
Source: FEBS Open Bio. 2022 Jul 11;12(8):1498–508. doi: 10.1002/2211-5463.13461 (PMC9340873; doi:10.1002/2211-5463.13461)
Supplement: Supplementary file 1 — Fig. S1. The effect of ERK5 on Mer at the RNA level. (A) RNA levels of Mer in 0, 1000 and 5000 U·mL−1 groups. (B) RNA levels of Mer in 0 U·mL−1, BIX02189, 5000 U·mL−1 and 5000 U+ BIX02189 groups. (C) RNA levels of Mer in 0 U·mL−1, XMD8‐92, 5000 U·mL−1 and 5000 U+ XMD8‐92 groups. [file FEB4-12-1498-s001.zip › feb413461-sup-0001-FigureS1 Legend.docx]

Supplementary Figure1. The effect of ERK5 on Mer at the RNA level. (A) RNA levels of Mer in 0 U/ ml, 1000 U/ ml and 5000 U/ ml groups.  (B) RNA levels of Mer in 0U/ ml, BIX02189, 5000U/ ml and 5000U+ BIX02189 groups.(C) RNA levels of Mer in 0U/ ml, XMD8-92, 5000U/ ml and 5000U+ XMD8-92 groups.
